# Myoepithelioma-like tumor of the vulvar region: a case report in China and review of the literature

**DOI:** 10.1186/s13000-019-0923-0

**Published:** 2020-01-08

**Authors:** Yan Xu, Hui Gao, Jin-Li Gao

**Affiliations:** 1 Department of Pathology, East Hospital, Tongji University, 1800 Yuntai Road, Pudong New District, Shanghai, 200120 China; 2 Central Laboratory, East Hospital, Tongji University, Shanghai, China

**Keywords:** Myoepithelioma-like tumor of the vulvar region, MELTVR, vulva, INI1/SMARCB1

## Abstract

**Background:**

Myoepithelioma-like tumor of the vulvar region (MELTVR) is a recently described mesenchymal neoplasm which typically arising in vulvar regions of adult women.

**Case presentation:**

Here we report a case of a 65-year-old woman who presented with a 6-year history of subcutaneous mass in the vulvar region. The mass had recently increased in size continuously. Histologically, the tumor cells had an epithelioid to spindled shape. Epithelioid tumor cells proliferated singly or in a loosely cohesive manner with myxoid areas, while spindled tumor cells grew in diffuse sheets or storiform arrangements mainly in nonmyxoid areas. Immunohistochemically, the tumor cells were positive for vimentin, epithelial membrane antigen, calponin, and were partially mild to moderate positive for estrogen receptor, but completely negative for S100 protein, glial fibrillary acidic protein, CD34, desmin, SMA and cytokeratin. INI1/SMARCB1 expression was deficient. *EWSR1* and *FUS* genes were intact tested by fluorescence in situ hybridization analysis. Based on these findings, we diagnose this case as MELTVR. The patient remained relapse-free after the lesion was widely excised during 8 months follow-up.

**Conclusions:**

This disease should be included in the differential diagnostic list of vulvar tumors with epithelioid to spindled morphology. Recognition of its histopathological features and immunohistochemical reactivity will help to understand the tumor better.

## Background

Myoepithelioma-like tumor of the vulvar region (MELTVR) is a soft-tissue neoplasm that is rarely observed in clinical practice; however, its typical characteristics have been described in the literature, including the histological, immunohistochemical and molecular signatures. MELTVR was first described by Yoshida et al. in 2015 [1], who reported nine cases arising from the vulvar region with a uniform INI/SMARCB1- deficient immunohistochemical reactivity. In our report, we present a case of MELTVR that arose in the vulva of a middle-age woman, which was initially suspected to be a leiomyoma.

## Case presentation

A 65-year-old woman presented with 6-year history of subcutaneous nodule in the vulvar region and recently the mass obviously increased creating personal discomfort. She was then admitted to our hospital for treatment. Computed tomography (CT) of the pelvis showed a cystic solid mass (diameter 50 mm) in the perianal region, suggesting a benign leiomyoma. The patient had no prior history of malignancy. Then, the patient underwent wide excision. On surgery, one nodule was found to be located in the vulvar muscle space, measuring approximately 45 mm in maximum diameter. The tumor was well-defined without obvious capsule. No sign of local recurrence or metastatic disease was observed after the initial excision during an eight-months follow-up.

**Fig. 1:**
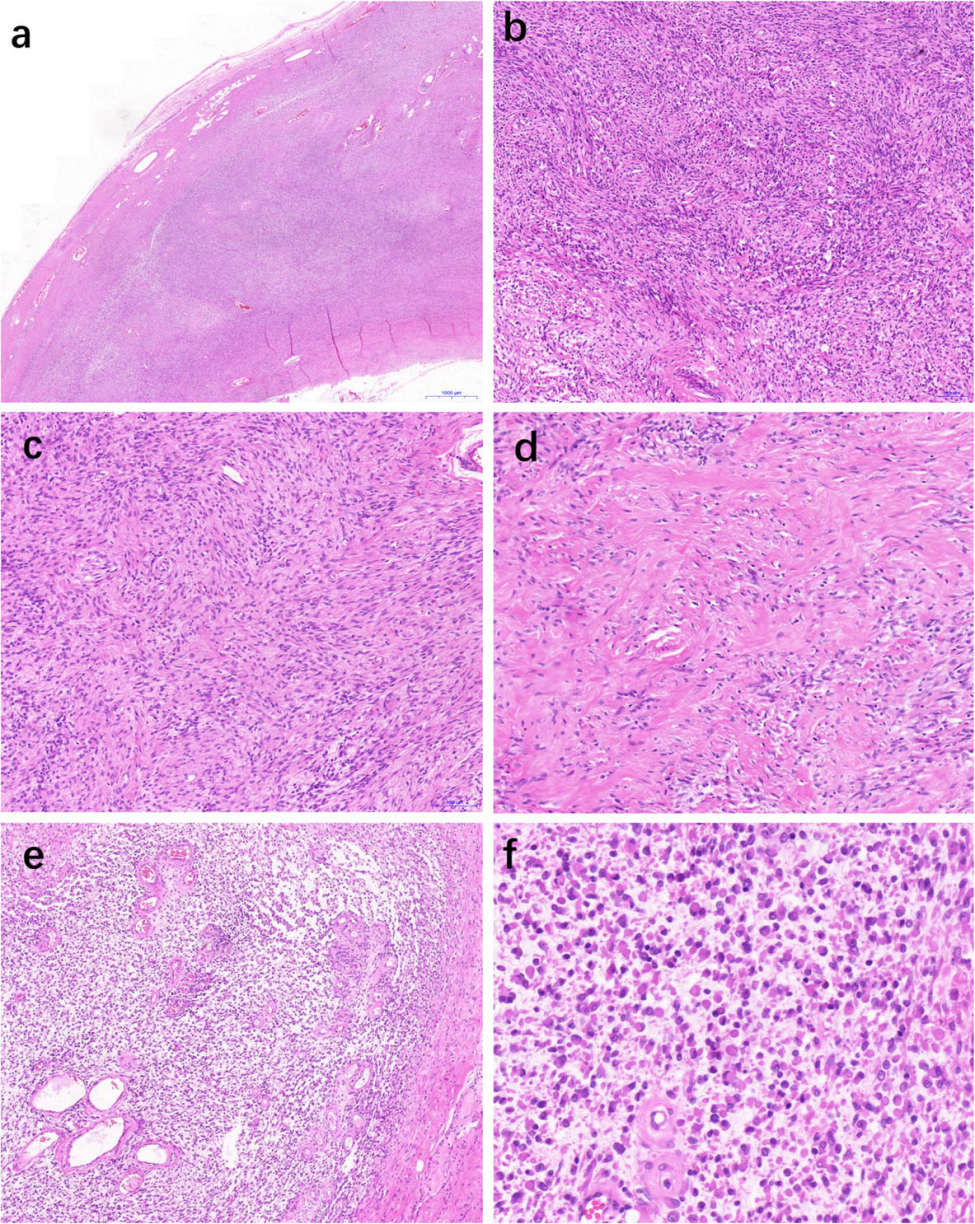
The tumor was a well-circumscribed nodule, focally encased by a fibrous pseudocapsule (**a**). In nonmyxoid patterns, the spindle-shaped tumor cells formed storiform arrangements, with a combination of hypocellular and hypercellular areas (**b**-**c**). The fibrous area shows deposition of hyalinizing collagen fibers (**d**). In myxoid patterns, epithelioid tumor cells proliferated singly or in a loosely cohesive reticular manner (**e**), In high power field, the epithelioid tumor cells resembled rhabdomyoblasts (**f**)

Grossly, the lesion was a well-circumscribed (Fig.1a), solid mass, with areas of translucent quality. On cut section, there was a solid white to gray lobulated nodule, measuring 5.5 × 4.0 × 3.5 cm in size. Histologically, at low magnification, the tumor was well circumscribed, focally encapsulated, and lobulated. The tumor stroma was relatively hypervascular and comprised a mixture of myxoid and nonmyxoid components, myxoid areas accounted for 20% of the tumor volume. At high magnification, the lesion was composed of spindle-shaped to epithelioid cells with abundant amphophilic cytoplasm, consisting of vesicular nuclei and small nucleoli. In nonmyxoid area, the tumors cells arranged in storiform pattern (Fig.1b, c) and some areas of tumor stroma was predominantly hyalinized or fibrous (Fig.1d). In myxoid areas, tumors cells grew singly or in a loosely cohesive manner with abundant eosinophilic cytoplasm resembling rhabdomyoblasts (Fig.1e, f). Rhabdomyoblasts-like cells accounted for approximately 80% of the total cells in the myxoid areas. The nuclear atypia was mild to moderate and mitotic figures were low (up to five mitosis per 50 high-power fields).

**Fig. 2:**
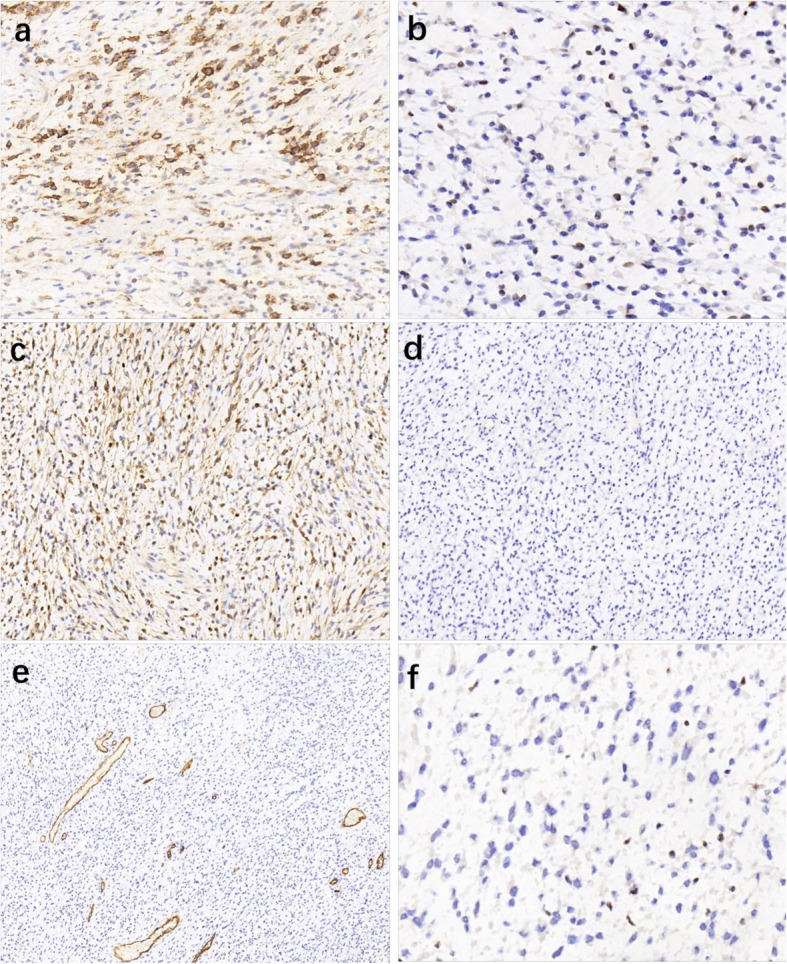
Immunohistochemical findings most tumor cells were partially and moderate positive for EMA (**a**), and mild to moderate positive for ER (**b**). Calponin was expressed both in the cytoplasm and in the nucleus of the tumor cells. (**c**). The tumor cells were completely negative for S-100(d) and CD34 (**e**). All tumor cells showed no INI1/SMARCB1 expression, while non-neoplastic endothelial cells expressed INI1/SMARCB1 (**f**)

Immunohistochemically, the tumor cells were diffusely positive for Vimentin, and partially positive for epithelial membrane antigen (EMA) with at least moderate intensity, which was mainly expressed in epithelioid cells (Fig.2a). Estrogen receptor (ER) was weakly expressed in some tumor cells (Fig.2b). Calponin (clone: CALP and EP63) was positive for both the nucleus and the cytoplasm of the tumor cells (Fig.2c). Focal expression for Bcl-2 and CD99 was observed. The tumor was negative for S100 protein (Fig.2d), cytokeratin (CK), glial fibrillary acidic protein (GFAP), CK7, SOX10, CD31, CD34 (Fig.2e), desmin, MyoD1, myogenin, smooth muscle actin (SMA), CD117, β-catenin and MUC4. Loss of INI1 protein expression was also confirmed (Fig.2f). The Ki67 index was about 10%. The results of immunochemical staining were summarized in Table 1.

**Table 1:** Immunohistochemical results of myoepithelioma-like tumor of the vulvar region

Antibody	Clone	Dilution	Source	Result
CK	AE1/AE3	1:100	Zhongshan china	–
CD34	10C9	1:100	Zhongshan china	–
S-100	4C4.9	Ready-to-use	Maixin China	–
GFAP	UMAB129	1:100	Zhongshan china	–
SMA	1A4	Ready-to-use	Maixin China	–
MyOD1	MX049	Ready-to-use	Maixin China	–
Myogenin	F5D	Ready-to-use	Maixin China	–
INI1	25	1:20	Zhongshan china	–
ER	SP1	1:300	Gene	+
Calponin	CALP	Ready-to-use	Maixin China	+
Calponin	EP63	Ready-to-use	Zhongshan china	+
vimentin	UMAB159	1:100	Zhongshan china	+
Bcl2	MX022	Ready-to-use	Maixin China	+
CD99	HO36–1.1	1:50	Zhongshan china	+
EMA	E29	Ready-to-use	Maixin China	+
CD31	UMAB30	1:100	Zhongshan china	–
KI-67	UMAB107	1:100	Zhongshan china	10%
DESMIN	EP15	1:100	Zhongshan china	–
MUC4	8G7	1:100	Zhongshan china	–
SOX10	EP268	1:100	Zhongshan china	–
B-catenin	UMAB15	1:100	Zhongshan china	–
CK7	OV-TL12/30	Ready-to-use	Maixin China	–
CD117	YR145	Ready-to-use	Maixin China	–

**Fig. 3:**
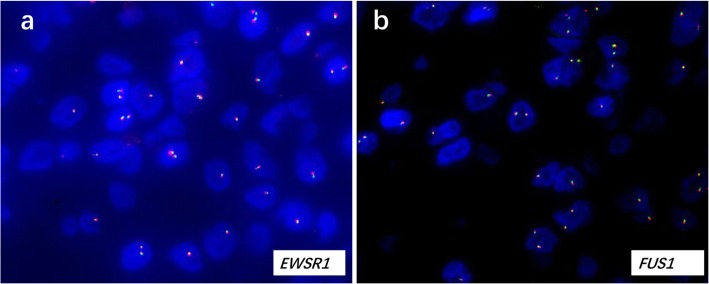
FISH results of present case. The tumor cells exhibited two pairs of fused signals by (**a**) EWSR1 and (**b**) FUS1 probes, no split signals were identified

Using formalin-fixed, paraffin-embedded 4-mm-thick tumor samples, dual-color break-apart fluorescence in situ hybridization (FISH) was used to investigate *EWSR1* and *FUS1* gene rearrangements. Break-apart probes for *EWSR1* (Abbott Molecular Inc., USA) and *FUS1* (Abbott Molecular Inc., USA) were used, and no split signals were observed with either probe (Fig.3a, b).

## Discussion

MELTVR is a rare neoplasm. Up to present, eleven cases of MELTVR have been reported in the literature [1–3]. The tumor is not classified according to the 4th edition of WHO classification of Soft Tissue and Bone Tumors [4]. MELTVR represents one of SMARCB1-deficient vulvar neoplasms [5]. Although it is difficult to diagnose the disease due to its rarity, it can be confirmed by the combination of histological and immunohistochemical features. In addition, molecular is also an important tool for differential diagnosis of MELTVRs and other tumors.

Based on the literatures, the clinical manifestation of MELTVR was not specific. Most patients presented with a painless mass or had occasional pain. The clinical diagnosis embraced a wide variety of disease, including solitary fibrous tumor, aggressive angiomyxoma, angiomyofibroblastoma, lipoma, hemangioma, and schwannoma [1, 2]. In our case, the lesion was originally considered leiomyoma or fibroma. At histology level, broad differential diagnoses need to be considered, including several tumors with loosely cohesive growths of epithelioid or spindle cells in a variable myxoid or hyalinized background. The morphology of this tumor resembles soft tissue myoepitheliomas, particularly those tumors with a myxoid pattern, but the neoplastic cells are negative for S100, GFAP and myogenic markers such as SMA, desmin. At molecular level, most of soft tissue myoepitheliomas harbor *EWSR1* gene translocation with a variety of different fusion partners, including *EWSR1*-*POU5F1*, *EWSR1*-*PBX1*, and *EWSR1*-*ZNF444* [6–8]. *FUS* gene rearrangements have also been reported in some myoepitheliomas [6, 9]. However, *EWSR1* and *FUS* rearrangements were absent in this tumor. The differential diagnosis of MELTVR also includes extraskeletal myxoid chondrosarcoma (EMC) due to its uniform, loosely cohesive tumor cells in a myxoid matrix. EMC is an extremely rare subtype of vulvar sarcoma that is consistently positive for vimentin, variable positivity for S100 protein, neuron-specific enolase and synaptophysin, completely negative for CK [10–16], and harhor *EWSR1-NR4A3* fusion in about 65% of cases [17]. However, the present case showed S100 negativity, EMA, ER positivity, and *EWSR1* was intact showed by FISH assay. Nevertheless, INI1/SMARCB1 expression has been retained in most EMC cases [18, 19], while the loss of INI1/SMARCB1 gene has also been reported in EMCs without major fusion gene transcript [18].

Due to the loss of expression of the INI1/SMARCB1, MELTVR need to be differentiated from INI1/SMARCB1-deficient vulvar neoplasms, including epithelioid sarcoma and extrarenal malignant rhabdoid tumor (E-MRT) [20–24]. Epithelioid sarcoma is a malignant tumor, which is divided into classical type of epithelioid sarcoma and proximal type of epithelioid sarcoma. The classical type of epithelioid sarcoma is often located in dermis. The tumor cells are relatively bland epithelioid cells, often showing a “granuloma-like” pattern of necrosis, which is easily misdiagnosed as rheumatoid nodules or annular granulomas [25]. The proximal type of epithelioid sarcoma typically shows a much greater degree of nuclear pleomorphism, more frequent rhabdoid cytology, and geographic necrosis [26], in contrast to the uniform nuclei and amphophilic cytoplasm of MELTVRs. Both forms of epithelioid sarcoma typically coexpress CAM5.2, AE1/AE3, EMA, CK19, vimentin and are CD34 positive in 50 to 70% of cases [27, 28]. However, AE1/AE3 and CD34 were completely negative in our case. E-MRT is a highly malignant small round cell tumor that occurs in infants and children. Cellular atypia was easily observed, and mitotic activity was high. E-MRT is extremely aggressive tumor, patients with this tumor often have a short survival [29, 30]. Unlike E-MRT, most MELTVRs occur in adult women, and the majority of MELTVRs showed an indolent clinical course. Immunohistochemically, E-MRT expressed AE1/AE3, CAM5.2, EMA and vimentin. Vimentin exhibited paranuclear globular staining, which was not observed in MELTVRs. In the present case, epithelioid cells with rhabdomyoblasts-like features in myxoid area was observed, which is needed to be differentiated from embryonal rhabdomyosarcoma (ERMS). However, ERMS is a highly malignant tumor with cellular atypia and a number of mitotic figures. Immunohistochemically, ERMS expresses SMA, desmin, MyoD1 and Myogenin, which are absent in MELTVRs.

The clinical and immunohistochemical features of the reported 11 cases and our case are summerized in Table 2. There is limited information regarding the incidence rate of MELTVRs due to its rarity. Immunohistochemically, there are no specific markers for this neoplasm, but the tumor cells are consistently positive for EMA and ER. All reported cases showed loss of INI1/ SMARCB1 expression, which appears to be a key factor to define the classification of this disease. In the present case, calponin was expressed both in the cytoplasm and in the nucleus of the tumor cells. We analyzed the phenomenon using two different clonal antibodies and the results are consistent. In addition, the present case showed a large number of rhabdomyoblasts-like cells which is not described. FISH analyses were negative for the presence of a rearrangement of the *FUS1* and *EWSR1* gene in our case. Biologically, all patients of reported cases survive without metastases, while three cases recurred after intralesional excision. Thus, we speculated that most of MELTVR showed an indolent clinical course. Our report is an important supplement to the morphology spectrum of MELTVR.

**Table 2:** Summary of clinical and immunohistochemical findings of previously reported and present cases of MELTVRs

Author	Age	Size (mm)	Immunohistochemical findings									R	M	Prognosis
			ER	EMA	GFAP	S-100	CD34	INI1	AE1/AE3	SMA	Desmin			(month)
Yoshida [1]	24–65	20–77	+	+	–	–	–	–	2/9*	5/8*	NA	3/9	–	1to172(mean,66) alive
Kaku [2]	31	20	+	+	–	–	–	–	+**	–	NA	–	–	11 m alive
Kojima [3]	70	36	+**	+	–	–	–	–	–	+	–	–	–	12 m alive
Present	65	55	+	+	–	–	–	–	–	–	–	–	–	8 m alive

*NA* data not available; *Rare (< 1%); **Focal (1 to 30%). *R* Recurrence; *M* Metastasis

## Conclusion

Based on previous reports and our observation, these tumors with low-grade malignant features and no metastases, wide excision and tumor-free margins seem to be an appropriate treatment. In summary, we have reported a rare case of MELTVR. Further investigation is required to clearly determine the pathological, immunohistochemical and molecular features of this tumor.

## Data Availability

All data generated or analyzed during this study are included in this published article.

## Abbreviations

CK: Cytokeratin
CT: Computed tomography
EMA: Epithelial membrane antigen
EMC: Extraskeletal myxoid chondrosarcoma
E-MRT: Extrarenal malignant rhabdoid tumor
ER: Estrogen receptor
ERMS: Embryonal rhabdomyosarcoma
FISH: Fluorescence in situ hybridization
MELTVR: Myoepithelioma-like tumor of the vulvar region
NSE: Neuron-specific enolase; Syn:synaptophysin
SMA: Smooth muscle actin
WHO: World Health Organization

## References

[1] Yoshida A, Yoshida H, Yoshida M (2015). Myoepithelioma-like tumors of the vulvar region: a distinctive group of SMARCB1-deficient neoplasms. Am J Surg Pathol.

[2] Kaku Y, Goto K, Kabashima K (2016). Myoepithelioma-like tumor of the vulvar region presenting as a Nonmyxoid spindle-cell neoplasm: a potential histologic mimicker of solitary fibrous tumor. Am J Dermatopathol.

[3] Kojima Y, Tanabe M, Kato I (2019). Myoepithelioma-like tumor of the vulvar region showing infiltrative growth and harboring only a few estrogen receptor-positive cells: a case report. Pathol Int.

[4] Fletcher CDMBJ, Hogendoorn PCW (2013). WHO classification of Tumours of soft tissue and bone.

[5] Folpe AL, Schoolmeester JK, McCluggage WG (2015). SMARCB1-deficient vulvar neoplasms: a Clinicopathologic, Immunohistochemical, and molecular genetic study of 14 cases. Am J Surg Pathol.

[6] Antonescu CR, Zhang L, Chang NE (2010). EWSR1-POU5F1 fusion in soft tissue myoepithelial tumors. A molecular analysis of sixty-six cases, including soft tissue, bone, and visceral lesions, showing common involvement of the EWSR1 gene. Genes Chromosomes Cancer.

[7] Brandal P, Panagopoulos I, Bjerkehagen B (2008). Detection of a t(1;22)(q23;q12) translocation leading to an EWSR1-PBX1 fusion gene in a myoepithelioma. Genes Chromosomes Cancer.

[8] Brandal P, Panagopoulos I, Bjerkehagen B, Heim S (2009). T(19;22)(q13;q12) translocation leading to the novel fusion gene EWSR1-ZNF444 in soft tissue myoepithelial carcinoma. Genes Chromosomes Cancer..

[9] Puls F, Arbajian E, Magnusson L, Douis H, Kindblom LG, Mertens F (2014). Myoepithelioma of bone with a novel FUS-POU5F1 fusion gene. Histopathology..

[10] Khan AS, Bakhshi GD, Shaikh A, Khan AA, Khan AA, Chitale A (2011). Extraskeletal chondrosarcoma of labium majus. Case Rep Pathol.

[11] Santacruz MR, Proctor L, Thomas DB, Gehrig PA (2005). Extraskeletal myxoid chondrosarcoma: a report of a gynecologic case. Gynecol Oncol.

[12] Sawada M, Tochigi N, Sasajima Y, Hasegawa T, Kasamatsu T, Kitawaki J (2011). Primary extraskeletal myxoid chondrosarcoma of the vulva. J Obstet Gynaecol Res.

[13] Villert A, Kolomiets L, Vasilyev N, Perelmuter V, Savenkova O (2015). Extraskeletal myxoid chondrosarcoma of the vulva: a case report. Oncol Lett.

[14] Hisaoka M, Hashimoto H (2005). Extraskeletal myxoid chondrosarcoma: updated clinicopathological and molecular genetic characteristics. Pathol Int.

[15] Okamoto S, Hisaoka M, Ishida T (2001). Extraskeletal myxoid chondrosarcoma: a clinicopathologic, immunohistochemical, and molecular analysis of 18 cases. Hum Pathol.

[16] Oliveira AM, Sebo TJ, McGrory JE, Gaffey TA, Rock MG, Nascimento AG (2000). Extraskeletal myxoid chondrosarcoma: a clinicopathologic, immunohistochemical, and ploidy analysis of 23 cases. Mod Pathol.

[17] Nishio J, Iwasaki H, Nabeshima K, Naito M (2011). Cytogenetics and molecular genetics of myxoid soft-tissue sarcomas. Genet Res Int.

[18] Kohashi K, Oda Y, Yamamoto H (2008). SMARCB1/INI1 protein expression in round cell soft tissue sarcomas associated with chromosomal translocations involving EWS: a special reference to SMARCB1/INI1 negative variant extraskeletal myxoid chondrosarcoma. Am J Surg Pathol.

[19] Le Loarer F, Zhang L, Fletcher CD (2014). Consistent SMARCB1 homozygous deletions in epithelioid sarcoma and in a subset of myoepithelial carcinomas can be reliably detected by FISH in archival material. Genes Chromosomes Cancer..

[20] Hollmann TJ, Hornick JL (2011). INI1-deficient tumors: diagnostic features and molecular genetics. Am J Surg Pathol.

[21] Hornick JL, Dal Cin P, Fletcher CD (2009). Loss of INI1 expression is characteristic of both conventional and proximal-type epithelioid sarcoma. Am J Surg Pathol.

[22] Orrock JM, Abbott JJ, Gibson LE, Folpe AL (2009). INI1 and GLUT-1 expression in epithelioid sarcoma and its cutaneous neoplastic and nonneoplastic mimics. Am J Dermatopathol.

[23] Hoot AC, Russo P, Judkins AR, Perlman EJ, Biegel JA (2004). Immunohistochemical analysis of hSNF5/INI1 distinguishes renal and extra-renal malignant rhabdoid tumors from other pediatric soft tissue tumors. Am J Surg Pathol.

[24] Judkins AR (2007). Immunohistochemistry of INI1 expression: a new tool for old challenges in CNS and soft tissue pathology. Adv Anat Pathol.

[25] Enzinger FM (1970). Epitheloid sarcoma. A sarcoma simulating a granuloma or a carcinoma. Cancer..

[26] Guillou L, Wadden C, Coindre JM, Krausz T, Fletcher CD (1997). "proximal-type" epithelioid sarcoma, a distinctive aggressive neoplasm showing rhabdoid features. Clinicopathologic, immunohistochemical, and ultrastructural study of a series. Am J Surg Pathol.

[27] Laskin WB, Miettinen M (2003). Epithelioid sarcoma: new insights based on an extended immunohistochemical analysis. Arch Pathol Lab Med.

[28] Folpe AL (2014). Selected topics in the pathology of epithelioid soft tissue tumors. Mod Pathol.

[29] Beckwith JB, Palmer NF (1978). Histopathology and prognosis of Wilms tumors: results from the first National Wilms' tumor study. Cancer..

[30] Haas JE, Palmer NF, Weinberg AG, Beckwith JB (1981). Ultrastructure of malignant rhabdoid tumor of the kidney. A distinctive renal tumor of children. Hum Pathol.

